# Enhancement of tanshinone production in *Salvia miltiorrhiza* hairy root cultures by metabolic engineering

**DOI:** 10.1186/s13007-019-0439-3

**Published:** 2019-05-23

**Authors:** Tao Wei, Yonghong Gao, Kejun Deng, Lipeng Zhang, Meiling Yang, Xiaopei Liu, Caiyan Qi, Chunguo Wang, Wenqin Song, Yong Zhang, Chengbin Chen

**Affiliations:** 1 National Pesticide Engineering Research Center (Tianjin), Nankai University, Tianjin, 300071 People’s Republic of China; 2 College of Life Sciences, Nankai University, Tianjin, 300071 People’s Republic of China; 3 School of Life Sciences and Technology, University of Electronic Science and Technology of China, Chengdu, 610054 People’s Republic of China; 4 Center for Informational Biology, University of Electronic Science and Technology of China, Chengdu, 610054 People’s Republic of China

**Keywords:** *Salvia miltiorrhiza*, Hairy roots, Tanshinones, *SmMDS.* elicitors

## Abstract

**Background:**

Tanshinones are diterpenoid compounds that are used to treat cardiovascular diseases. As current extraction methods for tanshinones are inefficient, there is a pressing need to improve the production of these bioactive compounds to meet increasing demand.

**Results:**

Overexpression of *SmMDS* (2-c-methyl-d-erythritol 2,4-cyclodiphosphate synthase, a tanshinone biosynthesis gene) in transgenic *Salvia miltiorrhiza* hairy roots significantly increased the tanshinone yield compared to the control, and total tanshinone content in *SmMDS*-overexpressing lines increased after elicitor treatment. Total tanshinones increased to 2.5, 2.3, and 3.2 mg/g DW (dry weight) following treatment with Ag^+^, YE (yeast extract), and MJ (methyl jasmonate), respectively, compared with the non-induced transgenic line (1.7 mg/g DW). Also, qRT-PCR analysis showed that the expression levels of two pathway genes was positively correlated with increased accumulation of tanshinone.

**Conclusions:**

Our study provides an effective strategy for increasing the content of tanshinones and other natural compounds using a combination of genetic engineering and elicitor treatment.

**Electronic supplementary material:**

The online version of this article (10.1186/s13007-019-0439-3) contains supplementary material, which is available to authorized users.

## Background

*Salvia miltiorrhiza* Bunge (Lamiaceae), also known as Chinese sage, is a traditional Chinese medicinal herb with remarkable medical and economic value that is widely used to treat menstrual, cardiovascular, and various inflammation-related diseases [1–5]. Tanshinones are bioactive diterpenoid compounds produced in roots of *S. miltiorrhiza* that have versatile pharmacological activities including antibacterial, antioxidant, anti-inflammatory, cardiovascular protective, and antineoplastic activities [6–10]. However, the low yield of tanshinones, which usually requires a large amount of plant material, has become a major obstacle to the further pharmaceutical development of *S. miltiorrhiza* [11–13].

The biosynthetic pathway of tanshinones has been well studied in *S. miltiorrhiza*, that are mainly derived from two common precursors, dimethylallyl diphosphate (DMAPP) and isoprene diphosphate (IPP) (Additional file 1: Fig. S1) [14–18]. These two precursors are synthesized in separate cell compartments by two different pathways; the MVA (mevalonate) pathway is found in the cytoplasm, while the MEP pathway is active in the plastid. The MEP (methylerythritol phosphate) pathway includes seven enzymatic reactions, starting from G3P and pyruvate, and is considered to be the main source for the biosynthesis of the C5 precursors of tanshinone [17, 19, 20]. IPP is a common intermediate in the two pathways, which is converted into diterpenoids by GGPPS (geranylgeranyl diphosphate synthase), CPS (copalyl diphosphate synthase), KSL (kaurene synthase-like), CPR (cytochrome P450 reductase) and several other unknown enzymes [3, 9, 17, 19]. According to the stated pathway, increased accumulation of the target metabolite can be achieved by elevating genes encoding key enzymes in the biosynthetic pathway or via the inhibition of gene expression in the competitive pathway in *S. miltiorrhiza* plants or hairy roots [12, 21, 22]. Compared with the control lines, production of tanshinone was significantly enhanced in transgenic hairy root lines overexpressing *SmGGPPS* and/or *SmHMGR* (hydroxymethylglutaryl-CoA reductase) as well as *SmDXS* (1-deoxy-d-xylulose 5-phosphate synthase) [21]. The co-expression of *SmGGPPS* and *SmHMGR* led to the highest yield of tanshinone in line HG9 (2.73 mg/g DW), which was significantly higher (5.7-fold) than the control (0.48 mg/g DW) [21]. MDS (2-c-methyl-d-erythritol 2,4-cyclodiphosphate synthase), which catalyzes the conversion of CDP-ME2P (2-phospho-4-(cytidine 5′-diphospho)-2-c-methyl-d-erythritol) to a cyclic intermediate MEcPP (2-c-methyl-d-erythritol 2,4-cyclodiphosphate) in the MEP pathway, has been successfully isolated from *S. miltiorrhiza* [17]. However, to date, there is no report on the effect of MDS gene on the accumulation of tanshinone in *S. miltiorrhiza*.

Hairy root cultures, which are induced by the naturally occurring soil bacterium *Agrobacterium rhizogenes* infects plant tissues [20, 23, 24], have been widely used to develop bioreactor processes suitable for hairy root cultures, to elucidate biosynthetic pathways, and to produce valuable plant-derived secondary metabolites [25–27]. Elicitors, which are defined as signaling compounds, can enhance or induce the biosynthesis of metabolites by activating pathways that respond to external stress [28, 29]. As with other in vitro culture systems, the accumulation of target metabolites can be readily increased by adding elicitors to hairy root cultures [30–32]. Previous studies have documented the effect of endogenous hormones and elicitors, such as salicylic acid (SA), methyl jasmonate (MJ), and yeast extract (YE), on tanshinone metabolism in hairy root cultures of *S. miltiorrhiza* [11, 22, 33].

Accordingly, we hypothesized that a combination of elicitor treatment and metabolic engineering might be a promising way to increase the yield of tanshinone. In the present study, manipulation of the MEP pathway (through over-expression of *SmMDS*) was carried out to increase tanshinone accumulation in *S. miltiorrhiza* hairy roots. To test whether the accumulation of tanshinone can be further increased by adding an elicitor to transgenic hairy roots, *SmMDS*-overexpressing lines were treated with different elicitors. The results of our study show that the combination of transgenic technology and elicitor treatment is an effective strategy to increase tanshinone levels in hairy root cultures, and potentially providing a way to improve tanshinones yields from current extraction procedures in hairy roots of *S. miltiorrhiza*.

## Methods

### Construction of plant expression vectors

The *SmMDS* open reading frame (ORF; accession No. JN831097.1) was PCR-amplified from an *S. miltiorrhiza* seedling (30 d) cDNA library using primers containing the restriction sites *Xba* I and *Xma* I at their 5′ ends (Additional file 6: Table S1). The PCR product (718 bp) was first cloned into the pMD18-T vector (TaKaRa Biotech). After digestion with *Xba* I and *Xma* I, the *SmMDS* ORF fragment was inserted into a modified pBI121 vector that contains a 2A peptide linked to the N-terminus of the GUS reporter gene, and the *NPT II* gene for kanamycin resistance as a selectable marker (Additional file 2: Fig. S2). The disarmed *Agrobacterium tumefaciens* strain C58C1 containing both the recombinant *SmMDS* binary vector plasmid and the *A*. *rhizogenes* Ri plasmid (pRiA4b) was used for plant genetic transformation. The C58C1 strain containing only Ri plasmid was used as transformation control.

### Plant transformation, molecular detection, and hairy root cultivation

**Fig. 1:**
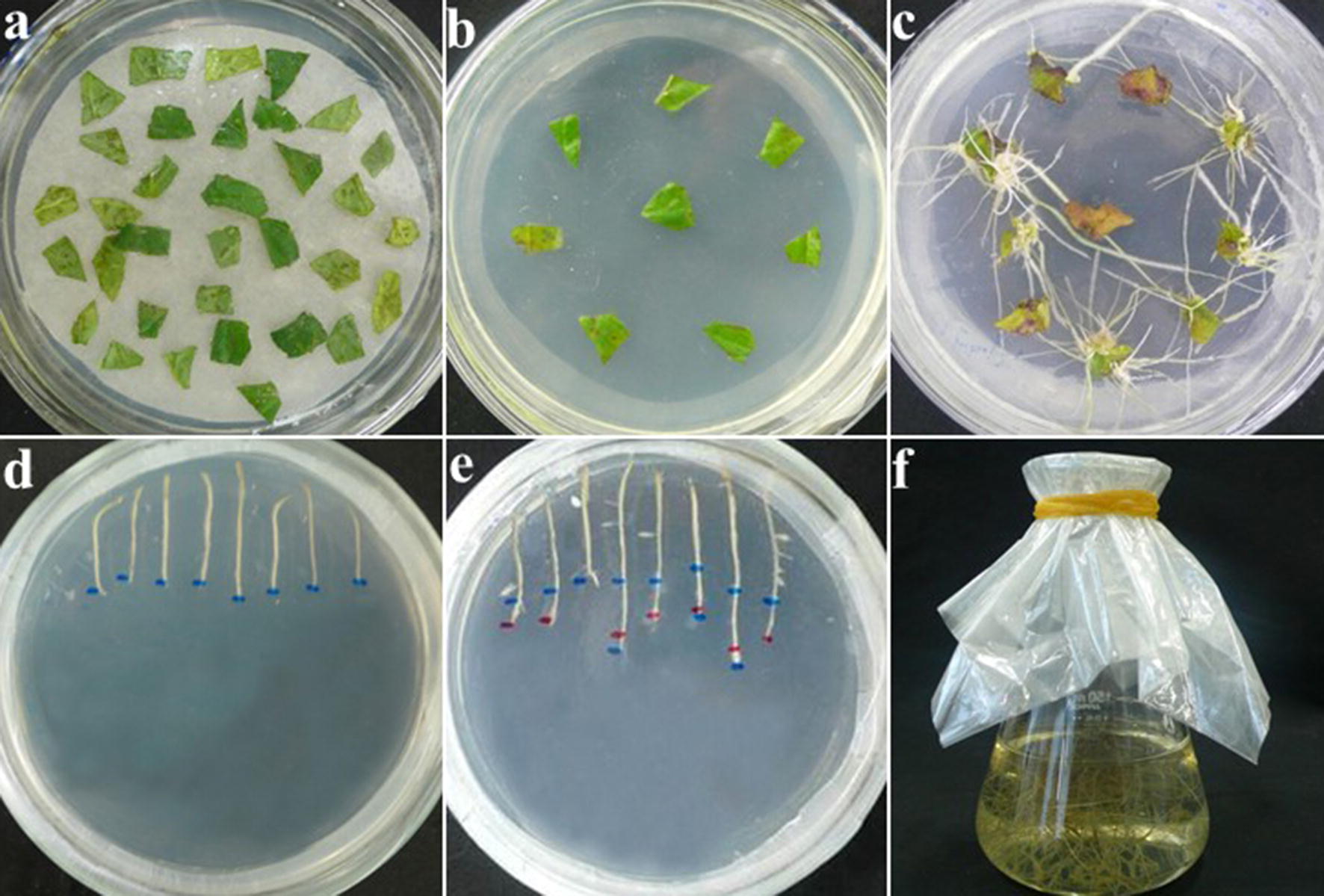
*Agrobacterium*-mediated transformation and transgenic hairy root proliferation in *Salvia miltiorrhiza*. **a** Tissue co-cultured with agrobacteria for 0 d; **b** induction culture at 0 d; **c** transgenic hairy roots proliferation after 20 d; **d** Antibiotic screening culture (400 mg/L Cef and 50 mg/L Kan) at 0 d; **e** Antibiotic screening culture (400 mg/L Cef and 50 mg/L Kan) after 6 d; **f** Liquid proliferation culture after 15 d

The aseptic *S. miltiorrhiza* plants were grown in half-strength Murashige and Skoog (0.5X MS) basal medium containing 3% sucrose and 0.7% agar (pH 5.8 ± 0.1), at 25 ± 1 °C with a 16 h light/8 h dark photoperiod. Leaves of aseptically-grown *S. miltiorrhiza* seedlings (30 d) were cut into 0.5 cm × 0.5 cm squares, infected with the *Agrobacterium* strain C58C1 for 25 min, and then co-cultured on MS medium with 100 µmol/L AS (acetosyringone) in the dark for 2-3 days. The infected leaves were transferred to 0.5X MS medium containing 400 mg/L Cef (cefotaxime sodium) and cultured for 2–3 weeks to control the Agrobacterium overgrowth. The hairy roots were then excised from the leaf pieces (2–3 cm in length) and transferred to B5 Medium containing 400 mg/L Cef and 50 mg/L Kan (kanamycin) for selecting transgenic hairy roots (Fig. 1). The wild-type hairy roots did not grow on the medium containing 50 mg/L Kan (Additional file 3: Fig. S3), so they were placed on a medium containing only 400 mg/L Cef. Hairy root lines were established using rapidly growing root lines that were free from bacterial contamination [30].

A modified cetyltrimethyl ammonium bromide (CTAB) method was used to extract genomic DNA from the kanamycin-resistant hairy roots [13]. The forward primer 35S-F and the reverse primer SmMDS-R, which were designed to the sequences of the CaMV 35S promoter and the *SmMDS* gene, respectively, were used to identify the p35S::*SmMDS* transgenic lines. Rol A-F and rol A-R primers were used to check the transformation with the *Agrobacterium rhizogenes* Ri plasmid. The sizes of these two amplification products were 746 bp and 304 bp, respectively. PCR amplification of an actin gene fragment (267 bp) was used as the internal control. The PCR-positive hairy roots were cut into approximately 4-cm-long sections and transferred to 150 mL Erlenmeyer flasks containing 100 mL of 0.5X B5 medium and grown on an orbital shaker at 150 rpm in the dark at 25 °C. The hairy root cultures were sub-cultured in 0.5X B5 medium every 28 days.

### GUS staining and analysis of gene expression by qRT-PCR

X-Gluc was used as a chromogenic substrate for histochemical localization of GUS enzyme activity [34]. A buffered sodium phosphate solution (50 mM, pH 7.2) containing 1 mM X-Gluc was used as the reaction mixture. The hairy root samples were incubated in the X-Gluc solution (containing 50 mM Potassium ferricyanide and 50 mM Potassium ferrocyanide) at 37 °C for 12 h, and the pigments were removed by clearing in 100% ethanol before observation.

Total RNA was extracted from hairy roots as described previously [35]. The integrity of the RNA was analyzed by 1.5% agarose gel electrophoresis (data not shown). Equal amounts of total RNA (2 µg) was used to synthesize cDNA using M-MLV reserve transcriptase (Promega) with a final concentration of 10 U/μL. qRT-PCR assays were performed on iCycler iQ5™ (Bio-Rad) real time PCR using the SYBR Green q-PCR kit. Each amplification reaction (20 μL) contained 10 μL of 2 × SYBR Green Mix, 1 μL of cDNA, and 0.25 μM of the forward and reverse primers. The amplification program was initiated with an initial denaturation step at 95 °C for 3 min, followed by 40 cycles of 30 s at 95 °C, 30 s at 60 °C and 30 s at 72 °C. The dissociation curve at the end of each run was used to monitor amplicon specificity. Relative gene expression levels were determined by using the 2^−ΔΔCT^ method and normalized to the expression of GAPDH and actin. All of the specific genes were analyzed three times under the same conditions.

### Elicitor preparation and elicitation

Elicitation was performed by treating the hairy roots with Ag^+^, YE, and MJ. AgNO_3_ was dissolved in distilled water at a concentration of 30 mmol/L and filter sterilized through a 0.22 μm membrane [36]. The yeast extract elicitor was made as described previously [28]. The MJ was first dissolved in a small amount of dimethylsulphoxide, then dissolved in distilled water to a storage concentration of 10 mmol/L, and sterilized through a 0.22 μm filter [33]. The elicitors were added to the shake flask cultures of *S. miltiorrhiza* hairy roots on day 60. The elicitor treatments were performed at the following concentrations: Ag^+^ 30 μmol/L, YE 100 mg/L, and MJ 100 μmol/L, and the hairy roots were collected for RNA extraction and tanshinone content analysis 45 days after treatment with the individual elicitors.

### Determination of tanshinone concentration by HPLC

After harvested from the shake flask cultures, the hairy roots were dried at 50 °C until a constant dry weight was reached. Tanshinone was isolated and detected according to previous report [37]. The dried samples were ground into powders and extracted with 80% methanol for 1 h in a constant temperature bath at 80 °C. The filtrates were analyzed on a Shimadzu LC-20AT high performance liquid chromatography (HPLC) system equipped with a C18 column (4.6 mm ID × 250 mm). The mobile phase consisted of gradient elution of solvent A (0.2% phosphoric acid) and B (acetonitrile). The flow rate was 0.7 mL/min, the detection wavelength was 270 nm, and the column temperature was 35 °C. For determination, the procedure was 0–25 min, solvent A (0.2% phosphoric acid) 15–30%, solvent B (acetonitrile), 85–70%; 25–30 min, solvent A, 30–60%, solvent B, 70–40%; 30–70 min, solvent A 60–80%, solvent B 40–20%. Based on the retention time and peak area, tanshinone was quantified by comparison with authentic standards. The total tanshinone content was calculated as the sum of tanshinone IIA, tanshinone I, cryptotanshinone, and dihydrotanshinone I.

### Statistical analysis

All reported data are expressed as the average value ± SD of at least three replicates. Statistical analyses (ANOVA) was performed using SAS software version 9.1 to test the significant differences between WT and transgenic or elicitor-induced hairy root lines. Duncan’s test (P < 0.05) was used to compare the mean value of each treatment group.

## Results

### Generation of transgenic hairy root lines

**Fig. 2:**
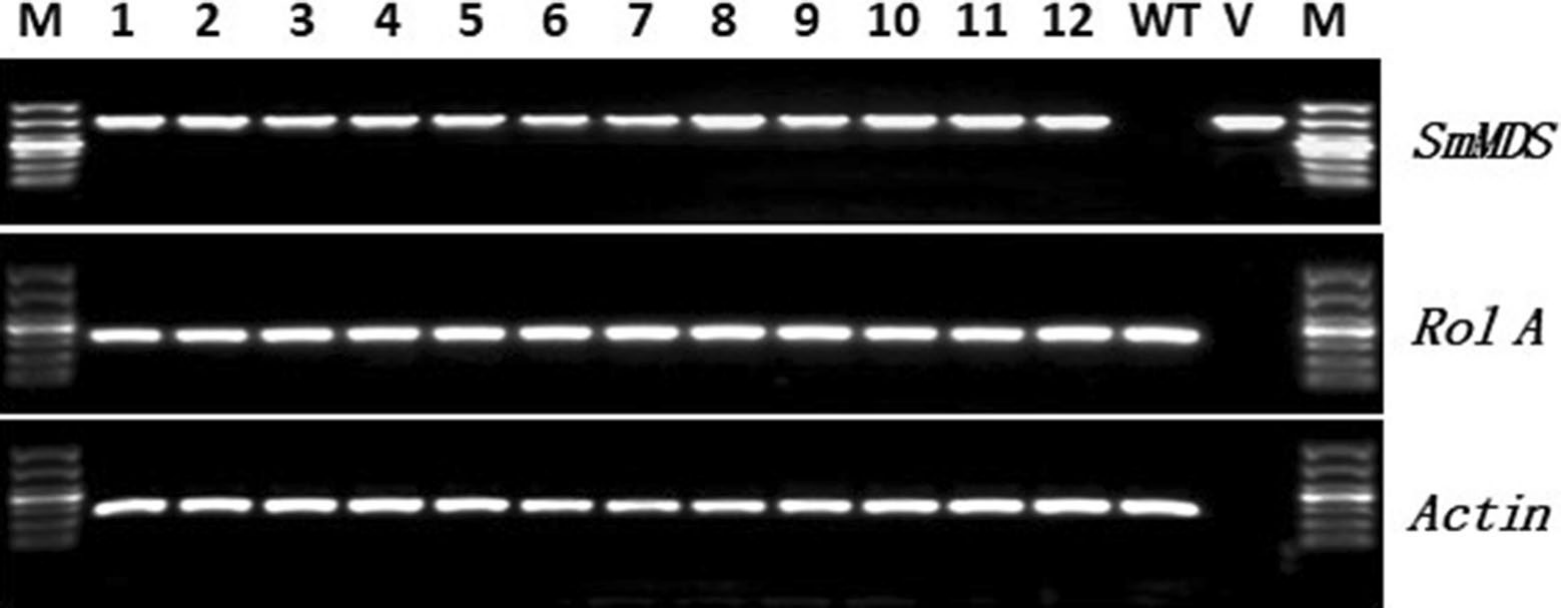
PCR analysis of primary transformants using primers specific to the 35S::*SmMDS*, rol A and actin genes. Lanes M, DNA size marker ladder; lanes 1–12, PCR products from putative transformants; lane WT, untransformed controls; lane V, DNA from plasmid p35S::SmMDS

**Fig. 3:**
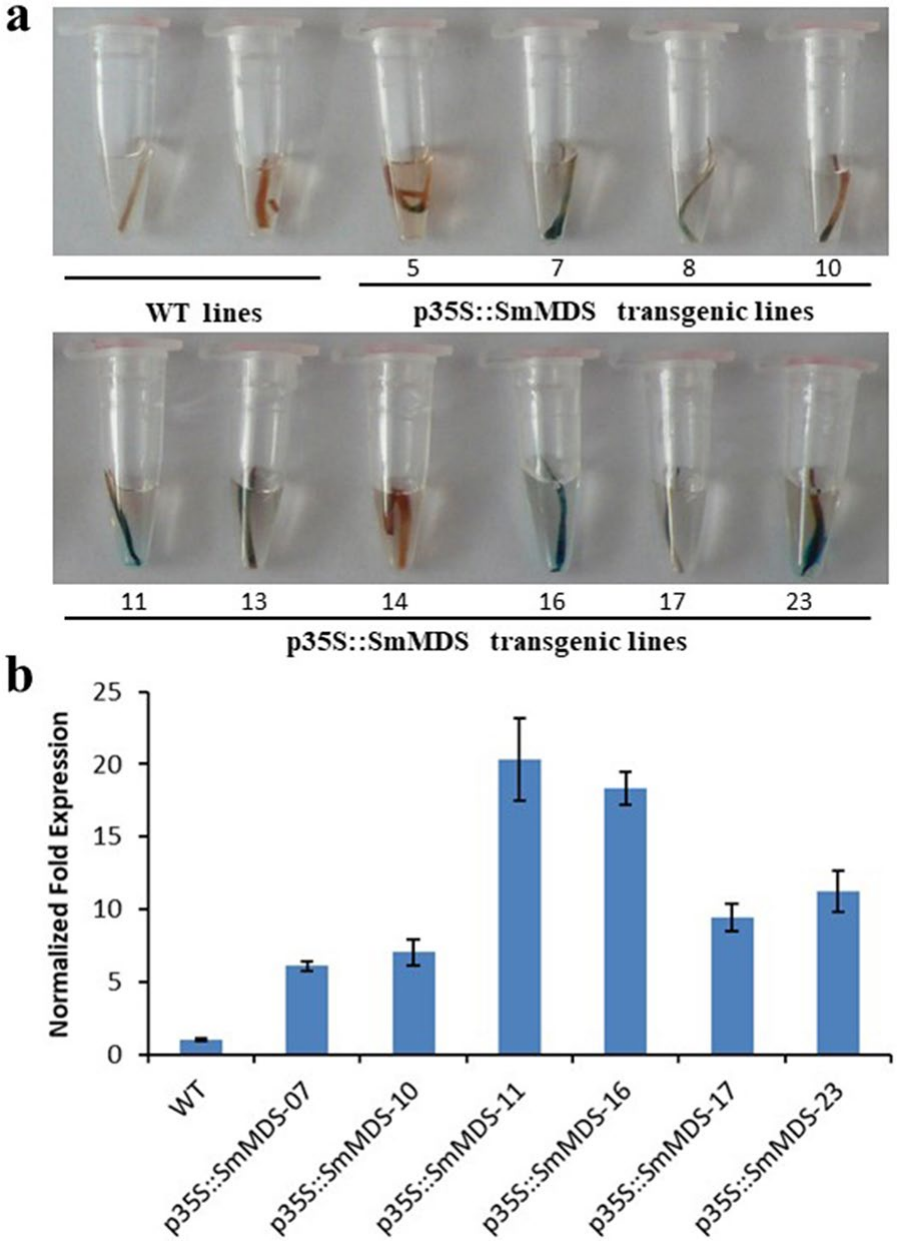
GUS staining (**a**) and expression of the *SmMDS* gene (**b**) in transgenic hairy roots (antibiotic screening culture for 15 d) of *S*. *miltiorrhiza*. WT, wild type; p35S::SmMDS-07, p35S::SmMDS transgenic line 07; p35S::SmMDS-10, p35S::SmMDS transgenic line 10; p35S::SmMDS-11, p35S::SmMDS transgenic line 11; p35S::SmMDS-16, p35S::SmMDS transgenic line 16; p35S::SmMDS-17, p35S::SmMDS transgenic line 17; p35S::SmMDS-23, p35S::SmMDS transgenic line 23. Values are presented as mean ± SD, n = 3. The y-axis in **b** shows the normalized expression pattern after log2 transformation

The vector containing the full-length *SmMDS* cDNA (Additional file 2: Fig. S2) was introduced into the modified *Agrobacterium* strain C58C1 which was then used to transform *S. miltiorrhiza* to produce transgenic hairy roots (Fig. 1). The hairy root lines that showed kanamycin resistance, a normal phenotype and normal growth,as WT hairy roots grow on kanamycin-free medium (data not shown), were used for further analysis. Genomic DNA was extracted from the selected *S. miltiorrhiza* hairy root lines and PCR analysis was performed using primers specifically designed to amplify the CaMV35S promoter and the N-terminal portion of the *SmMDS* gene. PCR primers rol A-F/R were used to identify hairy roots containing the *A. rhizogenes rol A* gene from *A*. *tumefaciens* strain C58C1 (Fig. 2). The PCR positive rate of the *SmMDS* hairy roots was 46/68 (67.6%). The GUS staining assays showed that the expression level of the *GUS* gene varied in the different hairy root lines (Fig. 3a). To investigate the expression of the introduced gene, we performed quantitative RT-PCR analysis of six independent transgenic lines that showed high levels of GUS expression (Fig. 3b). Two independent p35S::*SmMDS* transgenic hairy root lines (11 and 16) that expressed *SmMDS* at high levels were selected for further analysis.

### Morphology and biomass accumulation in the transgenic hairy roots

**Fig. 4:**
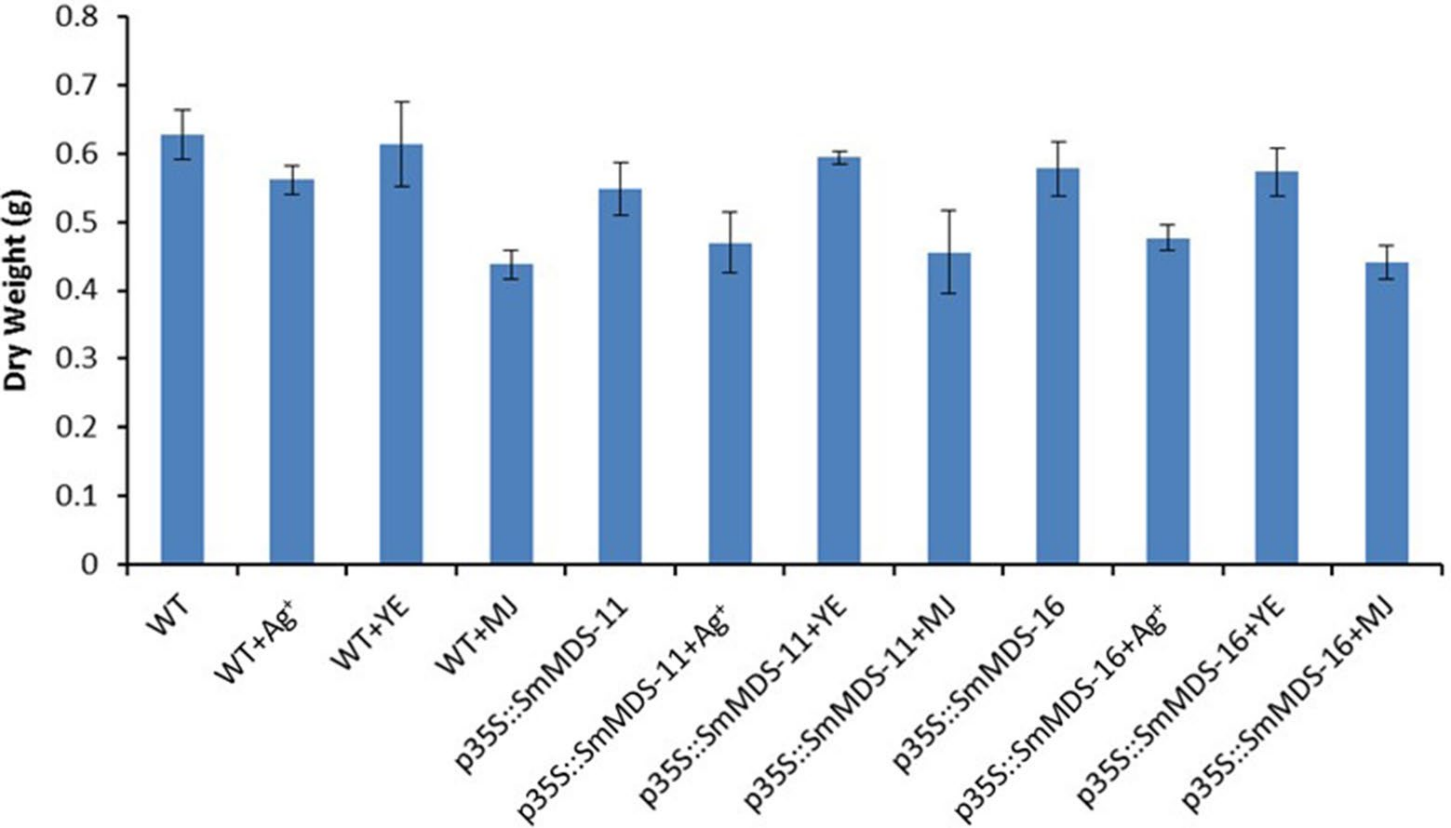
Dry weights of transgenic hairy roots on day 45 after elicitor treatments. WT, wild type; p35S::SmMDS-11, p35S::SmMDS transgenic line 11; p35S::SmMDS-16, p35S::SmMDS transgenic line 16. Values are presented as mean ± SD, n = 3

In order to obtain enough material for the assays, both wild-type and *p35S::SmMDS* transgenic hairy root lines were cultured in liquid medium. The *SmMDS*-expressing lines were slightly brown in color compared with the wild-type control after 60 days (Additional file 4: Fig. S4). To further enhance the production of tanshinone, elicitors were added to the control and the transgenic hairy root cultures on the 60th day after inoculation. After treatment for 45 days, the *SmMDS*-expressing lines, elicitor treated wild-type lines, and elicitor treated *SmMDS*-expressing lines were all darker brown in color than the wild-type lines. Among these, the color of the MJ-treated *SmMDS* transgenic lines was the darkest (Additional file 5: Fig. S5). We next measured the biomass of the hairy root cultures, which showed that the dry weights of the *SmMDS*-expressing lines, the elicitor-treated wild type lines, and the elicitor-treated *SmMDS*-expressing lines were all reduced, especially the MJ-treated lines, compared to the control (Fig. 4).

### Content of tanshinone in transgenic *S. miltiorrhiza* hairy roots

**Fig. 5:**
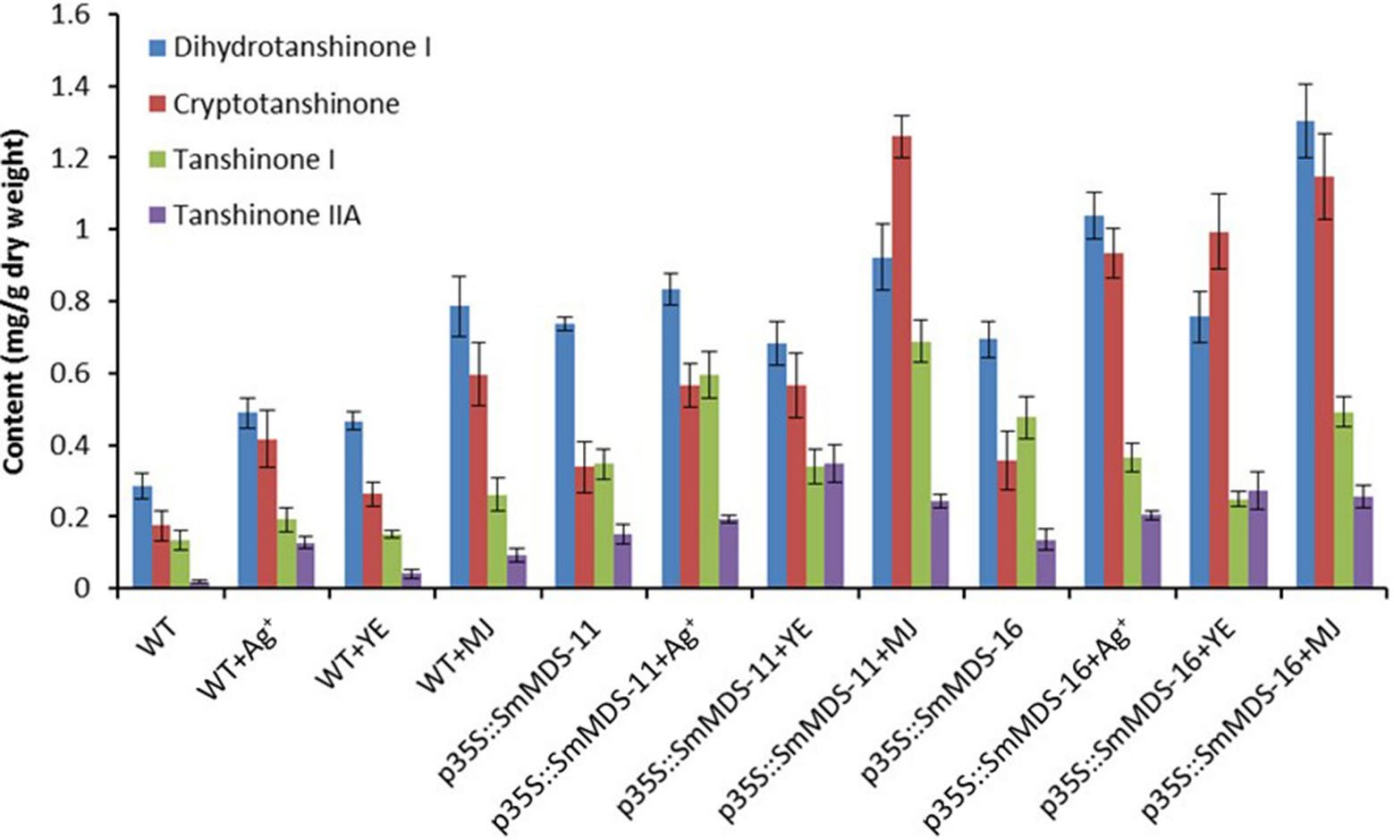
Contents of dihydrotashinone I, cryptotashinone, tanshinone I and tashinone IIA in transgenic hairy roots on day 45 after elicitor treatments. WT, wild type; p35S::SmMDS-11, p35S::SmMDS transgenic line 11; p35S::SmMDS-16, p35S::SmMDS transgenic line 16. Values are presented as mean ± SD, n = 3

**Fig. 6:**
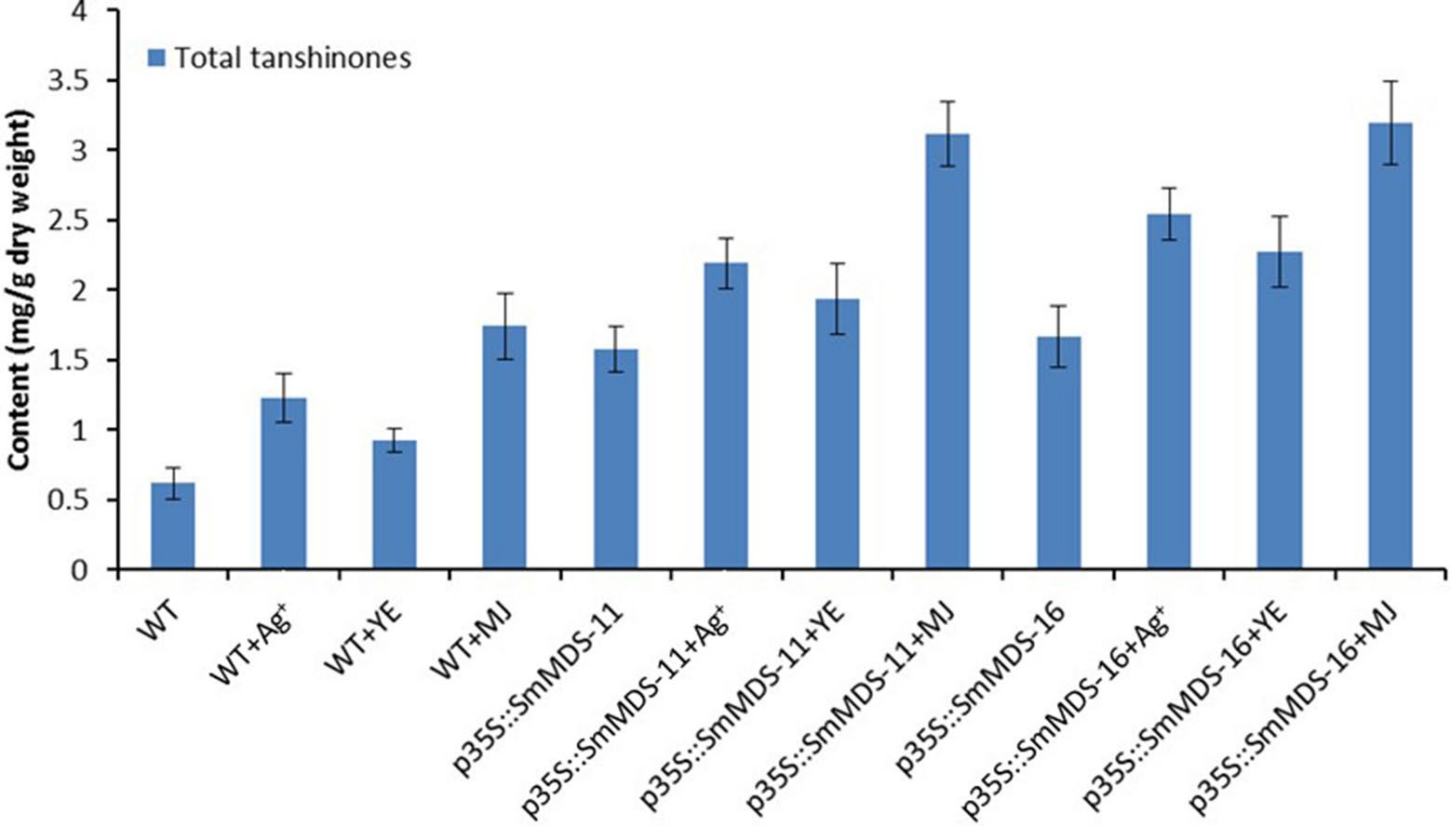
Total tanshinone production in transgenic hairy roots on day 45 after elicitor treatments. WT, wild type; p35S::SmMDS-11, p35S::SmMDS transgenic line 11; p35S::SmMDS-16, p35S::SmMDS transgenic line 16. Values are presented as mean ± SD, n = 3

To investigate the effects of over-expression *SmMDS* and/or elicitor treatment on the *S. miltiorrhiza* hairy root lines, the contents of tanshinone IIA, tanshinone I, dihydrotanshinone I, and cryptotanshinone were determined by high performance liquid chromatography (HPLC) (Fig. 5). The results showed that the MJ-treated p35S::*SmMDS*-16 line had the highest content of dihydrotanshinone I (1.3 mg/g DW), the MJ-treated p35S::*SmMDS*-11 line had the highest content of cryptotanshinone (1.26 mg/g DW) and tanshinone I (0.69 mg/g DW), the YE-treated p35S::*SmMDS*-11 line had the highest content of tanshinone IIA (0.35 mg/g DW). The sum of the four tanshinones was taken as the total tanshinone content. HPLC analysis revealed that, compared to the WT lines, the total tanshinone contents were significantly higher in the *SmMDS* lines, the elicitor-treated wild-type lines, and the elicitor treated *SmMDS* lines (Fig. 6). The MJ treated p35S::*SmMDS*-16 line had the highest content of total tanshinones (3.2 mg/g DW), 5.2-fold higher than that of the WT hairy root lines.

### Expression analysis of terpenoid biosynthetic genes after elicitor treatment

**Fig. 7:**
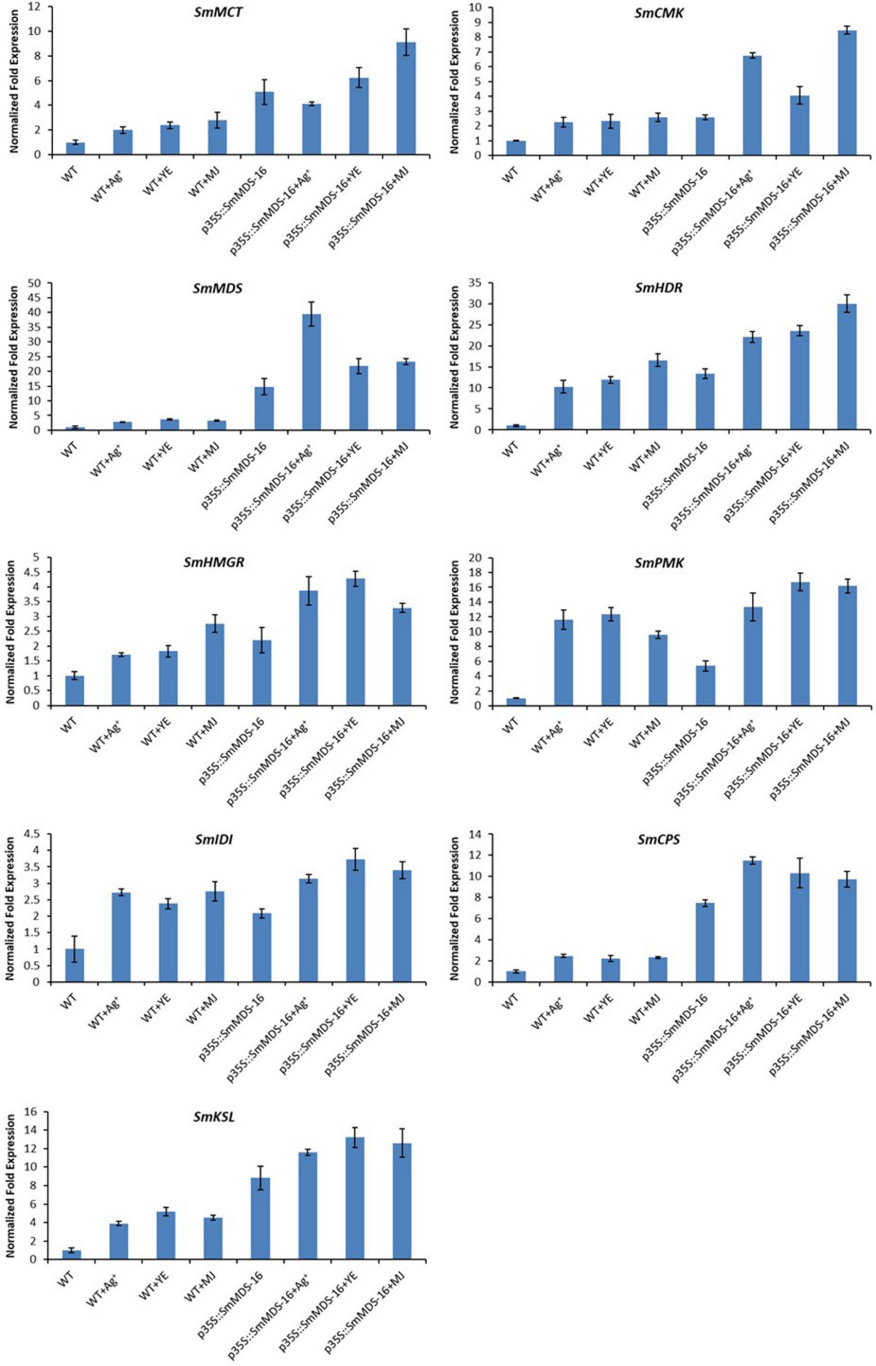
Relative expression of genes involved in tanshinone biosynthesis in transgenic hairy roots of *Salvia miltiorrhiza*. WT, wild type; p35S::SmMDS-11, p35S::SmMDS transgenic line 11; p35S::SmMDS-16, p35S::SmMDS transgenic line 16. Values are presented as mean ± SD, n = 3. The y-axis shows the normalized gene expression after log2 transformation. MCT, 2-c-methyl-d-erythritol 4-phosphate cytidylyltransferase; CMK, 4-(cytidine 5′-diphospho)-2-c-methyl-d-erythritol kinase; MDS, 2-c-methyl-d-erythritol 2,4-cyclodiphosphate synthase; HDR, 4-hydroxy-3-methylbut-2-enyl diphosphate reductase; HMGR, hydroxymethylglutaryl-CoA reductase; PMK, 5-phosphomevalonate kinase; IDI, isopentenyl diphosphate isomerase; CPS, copalyl diphosphate synthase; KSL, kaurene synthase-like

In order to further investigate the expression profiles of terpenoid pathway genes, we used the same samples that we had used for tanshinone quantification to conduct qRT-PCR assays with nine genes known to be related to the MEP and MVA pathways. As shown in Fig. 7, the relative expression levels of all nine genes were up-regulated in the *SmMDS* lines, elicitor treated wild-type lines, and the elicitor treated *SmMDS* lines. The highest relative expression levels of *SmMCT*, *SmCMK*, and *SmHDR* were detected in the MJ-treated p35S::*SmMDS*-16 line. The highest expression of *SmMDS* and *SmCPS* was in the Ag^+^-treated p35S::*SmMDS*-16 line. In the YE-treated p35S::*SmMDS*-16 line, we detected elevated expression of *SmHMGR*, *SmPMK*, *SmIDI*, and *SmKSL*. The above results indicate that the observed changes in tanshinone content and expression of genes in the tanshinone biosynthesis pathways were generally consistent.

## Discussion

Secondary metabolites with complex structures and remarkable activities found in plant cell suspension cultures and tissues have been extensively explored [38]. Hairy root cultures, which are transformed plant roots by Agrobacterium rhizogenes carrying the Ri T-DNA plasmid, have become the preferred biocatalyst over plant cell/callus and suspension cultures because of their biochemical/genetic stability, multi-enzyme synthesis potential, hormone-autotrophy, and relatively low-cost culture requirements [28, 39]. To date, hairy root cultures have been used to produce bioactive components of several medicinal plants [40, 41]. By successfully isolating tanshinone biosynthesis genes, such as *SmDXS*, *SmDXR*, *SmHMGR*, and *SmGGPPS*, from *S. miltiorrhiza*, attempts have been made to increase the yield of tanshinone in *S. miltiorrhiza* hairy roots by genetic engineering [22, 36, 42]. The tanshinone content in hairy roots overexpressing *SmHMGR* (H line) was found to be 1.567 mg/g, ~ 2.5-fold higher than in the control (0.613 mg/g) [36]. In our study, we report for the first time that overexpression of a single *MDS* gene in hairy roots of *S. miltiorrhiza* resulted in a large increase in the tanshinone content, indicating that MDS is a key regulatory point for regulating isoprene metabolism.

Because chemical elicitation is an effective strategy for increasing the biosynthesis of tanshinone, many abiotic (Ag^+^, Co^2+^, and Cd^2+^) and biotic elicitors (YE, MJ, and SA) have been previously used to treat hairy root cultures of *S. miltiorrhiza* [26, 43]. It has been widely reported that Ag^+^ can enhance the production of tanshinone in hairy roots of *S. miltiorrhiza* [44]. After treatment with 30 μM Ag^+^, the maximum content of total tanshinones in *S. miltiorrhiza* hairy roots was 1.2-fold higher than in the control [45]. In another study, 25 μM Ag^+^ increased the content of tanshinone I, cryptotanshinone, and tanshinone IIA by 0.87-, 30.0-, and 3.9-fold, respectively [43]. In the present study, Ag^+^ was also effective at inducing the production of the four tanshinone components (Fig. 5), and the contents of tanshinone I, tanshinone IIA, dihydrotanshinone I, and cryptotanshinone were 1.43-, 6.57-, 1.7-, and 2.38-fold higher than in the control, respectively. The total tanshinone content was doubled, which indicates that Ag^+^ is a potent elicitor of tanshinone production, and that tanshinone IIA is more sensitive to Ag^+^ elicitation than the other three tanshinone compounds.

Methyl jasmonate (MJ) and yeast extract (YE) are also considered to be effective elicitors that can increase the accumulation of tanshinone [22, 26]. Previous work has shown that, after treatment with 100 μM MJ, tanshinone levels increased after 3 days and reached a maximum of 0.93 mg/g DW on the 9th day, about 5.8-fold higher than the control (0.16 mg/g DW) [46]. The expression of many pathway genes was also shown to be up-regulated by MJ [46]. Yeast extract (YE) treatment can also increase the biosynthesis of tanshinone in hairy root cultures of *S. miltiorrhiza*, and the total content of tanshinones increased from 0.46 mg/g to 1.37 mg/g dry weight (DW) in a dose-dependent manner [47]. The contents of tanshinone IIA and cryptotanshinone (CT) were also increased by YE treatment. The results of a previous study showed that the accumulation of tanshinone increased after 3 days, and the level peaked at 0.643 mg/g DW on the 6th day, which was 3.99-fold higher than the control [46]. In our present study, tanshinone accumulation was also found to be induced by both MJ and YE treatment, and tanshinones increased by 2.8-fold and 1.5-fold, respectively, which was consistent with previous reports of elicitation.

Increased accumulation of tanshinones can be induced by co-overexpression of different pathway genes or a combination of different elicitors treatment. Co-overexpression of *SmDXSII* and *SmGGPPS* was shown to have a positive synergistic effect in stimulating the accumulation of plastid-derived isoprenoids and the diterpene tanshinone [42]. Cheng et al. reported that YE + Ag^+^ and YE + Ag^+^ + MJ were the most effective combinations of elicitors to stimulate accumulation of tanshinones, especially cryptotanshinone and dihydrotanshinone I, and the combined elicitors were more effective than individual elicitors at increasing tanshinone levels [28]. In addition, the combination of transgenic technology and elicitor treatment may be a viable strategy for tanshinone large-scale production in hairy roots of *S. miltiorrhiza* [33]. In this study, transgenic *SmMDS*-expressing hairy root lines were used for further elicitor treatment experiments. The maximum content of total tanshinones were significantly enhanced to 2.5, 2.3, and 3.2 mg/g DW by treatment with Ag^+^, YE, and MJ, respectively, compared with the non-induced p35S::*SmMDS*-16 line (1.7 mg/g DW), indicating that these elicitors have a significant effect on tanshinone accumulation of the transgenic *SmMDS* hairy root lines. Our results show that a combination of the two methods can improve tanshinone production in *S. miltiorrhiza* hairy root cultures more effectively than either genetic modification or elicitor treatment alone. In addition, *S. miltiorrhiza* hairy root culture has been proven to be a more effective alternative to farm growth of whole plants for tanshinone production [28]. In our previous research, the total tanshinones in the roots of *S. miltiorrhiza* plant was measured as 2.28 mg/g DW, after growth in an experimental field for 5 months [13], which was significantly lower than that of the MJ treated p35S::SmMDS-16 line (3.2 mg/g DW) on the 45th day in this study.

## Conclusion

Our research shows that combining transgenic technology with elicitor treatment (Ag^+^, YE, or MJ) can effectively increase the level of tanshinones in hairy root cultures of *S. miltiorrhiza*, making this an effective and feasible strategy for tanshinone production. Furthermore, our study also demonstrated that the accumulation of tanshinones is positively correlated with the expression levels of key biosynthesis genes in transgenic hairy roots of *S. miltiorrhiza*. The results presented here will pave the way for the metabolic engineering of tanshinone biosynthesis in hairy roots in the future.

## Additional files

**Additional file 1: Figure S1.** Biosynthesis of the diterpenoid tanshinones by the MEP and MVA pathways in the plastid and cytosol of *S. miltiorrhiza* [17].

**Additional file 2: Figure S2.** Diagram of the T-DNA region of the binary plasmid used for Agrobacterium tumefaciens-mediated transformation of *S. miltiorrhiza*.

**Additional file 3: Figure S3.** WT hairy roots on the selective medium (50 mg/L Kan) at 6 d.

**Additional file 4: Figure S4.** Morphology of transgenic hairy roots after cultured in liquid media for 60 days.

**Additional file 5: Figure S5.** Morphology of transgenic hairy root cultures on day 45 after elicitor treatments.

**Additional file 6: Table S1.** Oligonucleotide primers used in this study.

## Data Availability

The data generated or analyzed during this study are included in this published article and its supplementary information files.

## Abbreviations

MDS: 2-c-methyl-d-erythritol 2,4-cyclodiphosphate synthase
DW: dry weight
qRT-PCR: quantitative real time polymerase chain reaction
YE: yeast extract
MJ: methyl jasmonate
SA: salicylic acid
MEP: methylerythritol phosphate
MVA: mevalonate
IPP: isoprene diphosphate
GGPPS: geranylgeranyl diphosphate synthase
CPS: copalyl diphosphate synthase
KSL: kaurene synthase-like
CPR: cytochrome P450 reductase
G3P: glyceraldehyde 3-phosphate
ORF: open reading frame
CaMV: cauliflower mosaic virus
MS: Murashige and Skoog
HPLC: high-performance liquid chromatography
AS: acetosyringone
CTAB: cetyltrimethyl ammonium bromide
X-Gluc: 5-bromo-4-chloro-3-indole-glucuronide
DMAPP: dimethylallyl diphosphate
Cef: cefotaxime sodium
Kan: kanamycin
