# Supplementary material for: Enhancement of tanshinone production in Salvia miltiorrhiza hairy root cultures by metabolic engineering
Source: Plant Methods. 2019 May 23;15:53. doi: 10.1186/s13007-019-0439-3 (PMC6532201; doi:10.1186/s13007-019-0439-3)
Supplement: Supplementary file 1 — Additional file 1: Figure S1. Biosynthesis of the diterpenoid tanshinones by the MEP and MVA pathways in the plastid and cytosol of S. miltiorrhiza [17]. [file 13007_2019_439_MOESM1_ESM.docx]

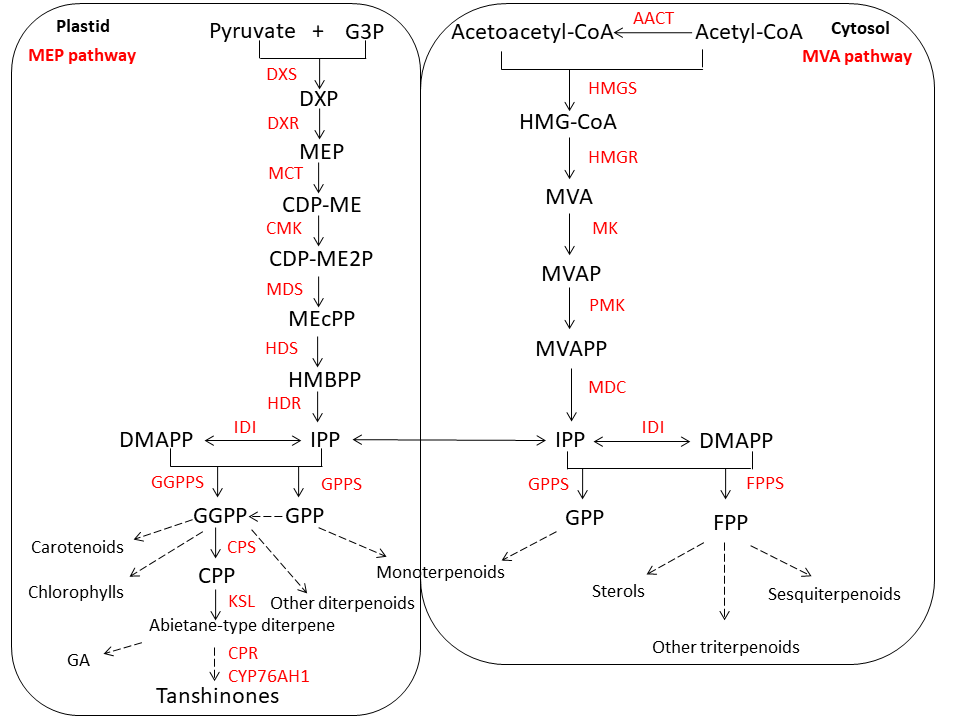


**Additional file 1: Figure S1. Biosynthesis of the diterpenoid tanshinones by the MEP and MVA pathways in the plastid and cytosol of *S. miltiorrhiza* [17].**
