# Supplementary material for: Enhancement of tanshinone production in Salvia miltiorrhiza hairy root cultures by metabolic engineering
Source: Plant Methods. 2019 May 23;15:53. doi: 10.1186/s13007-019-0439-3 (PMC6532201; doi:10.1186/s13007-019-0439-3)
Supplement: Supplementary file 2 — Additional file 2: Figure S2. Diagram of the T-DNA region of the binary plasmid used for Agrobacterium tumefaciens-mediated transformation of S. miltiorrhiza. [file 13007_2019_439_MOESM2_ESM.docx]

**
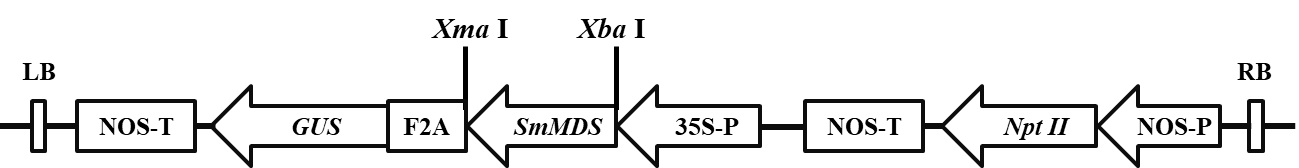
**

**Additional file 2: Figure S2. Diagram of the T-DNA region of the binary plasmid used for Agrobacterium tumefaciens-mediated transformation of *S. miltiorrhiza*.** The p35S::SmMDS binary plasmid containing *SmMDS* and *GUS* under the control of the 35S promoter，*NPT Ⅱ* under the control of the neomycin phosphotransferase promoter. 35S-P, the CaMV 35S RNA promoter; *NOS*-P, the promoter region of nopaline synthase; *NOS*-T, the 3' nopaline synthase terminator region; *NPT II*, the neomycin phosphotransferase gene; F2A, self-cleaving 2A peptide; LB, left T-region border; RB, right T-region border.
