# Supplementary figures and images for: Enhancement of tanshinone production in Salvia miltiorrhiza hairy root cultures by metabolic engineering
Source: Plant Methods. 2019 May 23;15:53. doi: 10.1186/s13007-019-0439-3 (PMC6532201; doi:10.1186/s13007-019-0439-3)

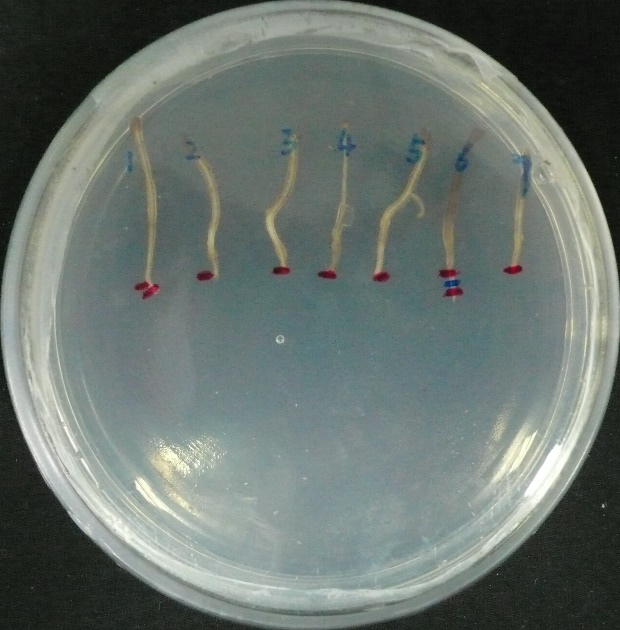


**Additional file 3: Figure S3.** **WT hairy roots on the selective medium (50 mg/L Kan) at 6 d.**

Supplement: Supplementary file 3 — Additional file 3: Figure S3. WT hairy roots on the selective medium (50 mg/L Kan) at 6 d. [file 13007_2019_439_MOESM3_ESM.docx]
