# Supplementary material for: Enhancement of tanshinone production in Salvia miltiorrhiza hairy root cultures by metabolic engineering
Source: Plant Methods. 2019 May 23;15:53. doi: 10.1186/s13007-019-0439-3 (PMC6532201; doi:10.1186/s13007-019-0439-3)
Supplement: Supplementary file 4 — Additional file 4: Figure S4. Morphology of transgenic hairy roots after cultured in liquid media for 60 days. [file 13007_2019_439_MOESM4_ESM.docx]

**
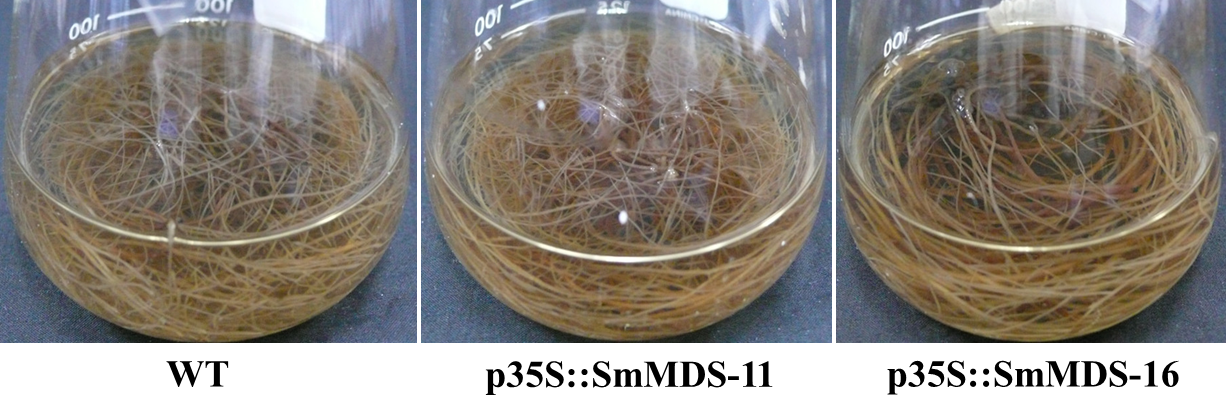
**

**Additional file 4: Figure S4. Morphology of transgenic hairy roots after cultured in liquid media for 60 days.** WT, wild type; p35S::SmMDS-11, p35S::SmMDS transgenic line 11; p35S::SmMDS-16, p35S::SmMDS transgenic line 16.
