# Supplementary material for: Enhancement of tanshinone production in Salvia miltiorrhiza hairy root cultures by metabolic engineering
Source: Plant Methods. 2019 May 23;15:53. doi: 10.1186/s13007-019-0439-3 (PMC6532201; doi:10.1186/s13007-019-0439-3)
Supplement: Supplementary file 6 — Additional file 6: Table S1. Oligonucleotide primers used in this study. [file 13007_2019_439_MOESM6_ESM.docx]

**Additional file 6: Table S1. Oligonucleotide primers used in this study.**

| **Oligo name** | **Sequence (5’ to 3’)** |  | **Oligo name** | **Sequence (5’ to 3’)** |  | **Oligo name** | **Sequence (5’ to 3’)** |
| --- | --- | --- | --- | --- | --- | --- | --- |
| SmMDS-F | TCTAGAATGGCTATGGCTGGTTCC |  | Rol A-F | CATGTTTCAGAATGGAATTA |  | Actin-F | GGTGCCCTGAGGTCCTGTT |
| SmMDS-R | CCCGGGCTTCCTAAAAAGGAGAAC |  | Rol A-R | AGCCACGTGCGTATTAATCC |  | Actin-R | AGGAACCACCGATCCAGACA |
| GAPDH-F | CCACCGTCCACTCCATCACT |  | GAPDH-R | TGGGAACTCGGAACGACATAC |  | 35S-F | GTTCATTTCATTTGGAGAGAACACG |
| Primers for detection of expression levels of biosynthetic pathway genes | | | | | | | |
| MCT-F | GGGTGTTGGGTGTTCCTGCTA |  | CMK-F | ACCGTGGCTCCTCGTCTTTAC |  | MDS-F | GGAGGAGGCAGTTCGGCTAAT |
| MCT-R | GGGCGTCATCGGTAACTTCG |  | CMK-R | CGGAATCCCAGCATCCCTAT |  | MDS-R | AGAGGTTCACAACGGAAGGGTC |
| HDR-F | GCATTGGCGGATGGAACTC |  | HMGR-F | TGAGGCTGCAAGGCAATCTATG |  | PMK-F | GGGTTCTGCTGGCTGGTGT |
| HDR-R | CCCTCTTTCTCCACCAACTCG |  | HMGR-R | GCTTCAACTCTGTCGCCCTCTT |  | PMK-R | CCGAGGGTCGTTGCTTTCTA |
| IDI-F | GGTTGTCGTCCTTGACCAGC |  | CPS-F | TGGATGGGCAGCAGCAGTA |  | KSL-F | GGGTGATTACTTGTCTGCCTCC |
| IDI-R | GCGTTGAGCGGAGAAATCG |  | CPS-R | GCGGCGACACGCTTATTC |  | KSL-R | GGGTTTTCTTTGCGTTCCTTC |
